# A narrative review for platelets and their RNAs in cancers: New concepts and clinical perspectives

**DOI:** 10.1097/MD.0000000000032539

**Published:** 2022-12-30

**Authors:** Yunhui Xiang, Pinpin Xiang, Liuyun Zhang, Yanying Li, Juan Zhang

**Affiliations:** a Department of Laboratory Medicine and Sichuan Provincial Key Laboratory for Human Disease Gene Study, Sichuan Provincial People’s Hospital, University of Electronic Science and Technology of China, Chengdu, China; b Department of Laboratory Medicine, Xiping Community Health Service Center of Longquanyi District Chengdu City, Chengdu, China.

**Keywords:** diagnostics, platelet-educated tumor cells, RNA, tumor microenvironment, tumor-educated platelets (TEPs)

## Abstract

Recent years have witnessed a growing body of evidence suggesting that platelets are involved in several stages of the metastatic process via direct or indirect interactions with cancer cells, contributing to the progression of neoplastic malignancies. Cancer cells can dynamically exchange components with platelets in and out of blood vessels, and directly phagocytose platelets to hijack their proteome, transcriptome, and secretome, or be remotely regulated by metabolites or microparticles released by platelets, resulting in phenotypic, genetic, and functional modifications. Moreover, platelet interactions with stromal and immune cells in the tumor microenvironment lead to alterations in their components, including the ribonucleic acid (RNA) profile, and complicate the impact of platelets on cancers. A deeper understanding of the roles of platelets and their RNAs in cancer will contribute to the development of anticancer strategies and the optimization of clinical management. Encouragingly, advances in high-throughput sequencing, bioinformatics data analysis, and machine learning have allowed scientists to explore the potential of platelet RNAs for cancer diagnosis, prognosis, and guiding treatment. However, the clinical application of this technique remains controversial and requires larger, multicenter studies with standardized protocols. Here, we integrate the latest evidence to provide a broader insight into the role of platelets in cancer progression and management, and propose standardized recommendations for the clinical utility of platelet RNAs to facilitate translation and benefit patients.

## 1. Introduction

The strong genetic and phenotypic heterogeneity and constant evolution of tumor cells pose challenges for early diagnosis and treatment based on traditional solid biopsies.^[1,2]^ Meanwhile, precision medicine requires more from biomarkers, such as molecular subtype classification, clonal evolution tracking, and treatment response monitoring.^[3]^ However, the application of liquid biopsies, such as circulating tumor deoxyribonucleic acid, circulating tumor cells, extracellular vesicles, and microRNAs (miRNAs), in the early diagnosis of cancer is also restricted by their nonspecific origin, physiological variables, and technical limitations, and more reliable biomarkers are urgently needed.^[3–5]^

It is now well understood that platelets have a bidirectional interaction with tumor cells, influencing several steps of tumor progression through a variety of components.^[6]^ Best et al demonstrated that tumors “educate” platelets by altering the platelet ribonucleic acid (RNA) profile, which has the potential for cancer diagnosis, pioneering platelet RNA as a liquid biopsy analyte.^[7]^ Simultaneously, platelets can efficiently transfer membrane lipids, proteins, and RNAs to “educate” tumor cells, referred to as platelet-educated tumor cells, granting them highly dynamic and aggressive phenotypes such as epithelial–mesenchymal transition (EMT), stem cell-like phenotypes, and highly proliferative capability.^[8]^

As research continues, multiple classes of platelet RNA have come to light, including messenger RNAs (mRNAs), miRNAs, circular RNAs (circRNAs), long non-coding RNAs (lncRNAs), YRNAs, and exogenous non-coding RNAs, possibly derived from the environment.^[9]^ A 4-year longitudinal study found that platelet RNA expression in healthy individuals is generally stable across and within individuals, except for a subset of genes enriched in the inflammatory process, a property that would facilitate its clinical application.^[10]^ Platelet RNA analysis is expected to reveal the latest, enhanced, and dynamic reflection of tumor activity, as tumor-derived transcripts accumulate and are protected in platelets that normally live for only 7 to 10 days, making it possible to monitor tumor progression and adjust treatment plans in time.^[11]^ However, platelets have a wide range of effects on the tumor microenvironment, and the underlying molecular mechanisms remain elusive.^[12]^ Furthermore, the utility of platelet RNAs in tumor diagnosis, prognosis, and guiding treatment remains controversial.^[13–15]^ Hence, this review aimed to summarize the complex roles and possible clinical applications of platelets and their RNAs in cancer with the latest evidence.

## 2. Platelets and their RNAs interact with cancer cells

### 2.1. Platelets and tumor cells exchange cellular components inside and outside the blood vessels

Accumulating evidence from clinical and animal studies supports the view that platelets are essential and active members of the tumor microenvironment rather than incidental bystanders. Rodriguez-Martinez et al found that biological interactions between platelets and several types of cancer cells occur through direct contact, platelet phagocytosis, or via microparticles.^[8]^ Phagocytosis of platelets by cancer cells in a dynamin-dependent manner was also observed by the team of Martins Castanheira, allowing cancer cells to access the entire platelet proteome, transcriptome, and secretome. Platelet uptake was not observed in the non-cancer cell line 16HBE14o-, implying that platelet uptake may be a feature of cancer cells but needs to be confirmed by additional studies in more types of cancer and non-cancer cells.^[16]^

Scientists have long recognized that tumor-platelet interactions can be mediated by tumor- and platelet-derived microparticles (PMP). Tumor-derived microparticles shed into the blood can activate platelets via soluble procoagulant factors such as tissue factor, thrombin, and adenosine diphosphate, promoting the formation of microclots and protective cloaks around circulating tumor cells, protecting them from shear force and immune cell attack, which is vital for their survival.^[17–19]^ Recently, Liu et al reported that targeted silencing of tumor tissue factors inhibits metastasis and prevents cancer-related hypercoagulation by suppressing the formation of thrombin-antithrombin complexes and activation of platelets.^[20]^ PMPs are shed from the platelet membrane and contain platelet-derived CD41 and CD62p. Sustained release of PMP can occur during platelet homeostasis and is enhanced in response to numerous stimuli such as complement proteins, thrombin, and shear force. PMPs are the most abundant source of plasma-borne microparticles, which carry bioactive lipids, proteins, genetic materials, and organelles from their parental platelets. Recently, Plantureux et al demonstrated that platelets interact with tumor cells in a cadherin-6-dependent manner, resulting in the production of 3 different types of microparticles in colorectal cancer tissue that have platelet markers, tumor markers, or both, collectively known as platelet- and tumor cell-interacting microparticles (iMPs). iMPs significantly suppressed tumor growth by enhancing the recruitment of intratumoral macrophages through the chemoattractants regulated upon activation normal t-cell expressed and secreted, MIF, CCL2, and CXCL12, and activation of their tumoricidal capacity via interferon gamma (IFNγ) and interleukin (IL)-4. However, iMPs can also support metastasis through EMT and endothelial cell activation. These balance tilts may hinge on the environment, local or blood flow.^[21]^

**Figure 1.**
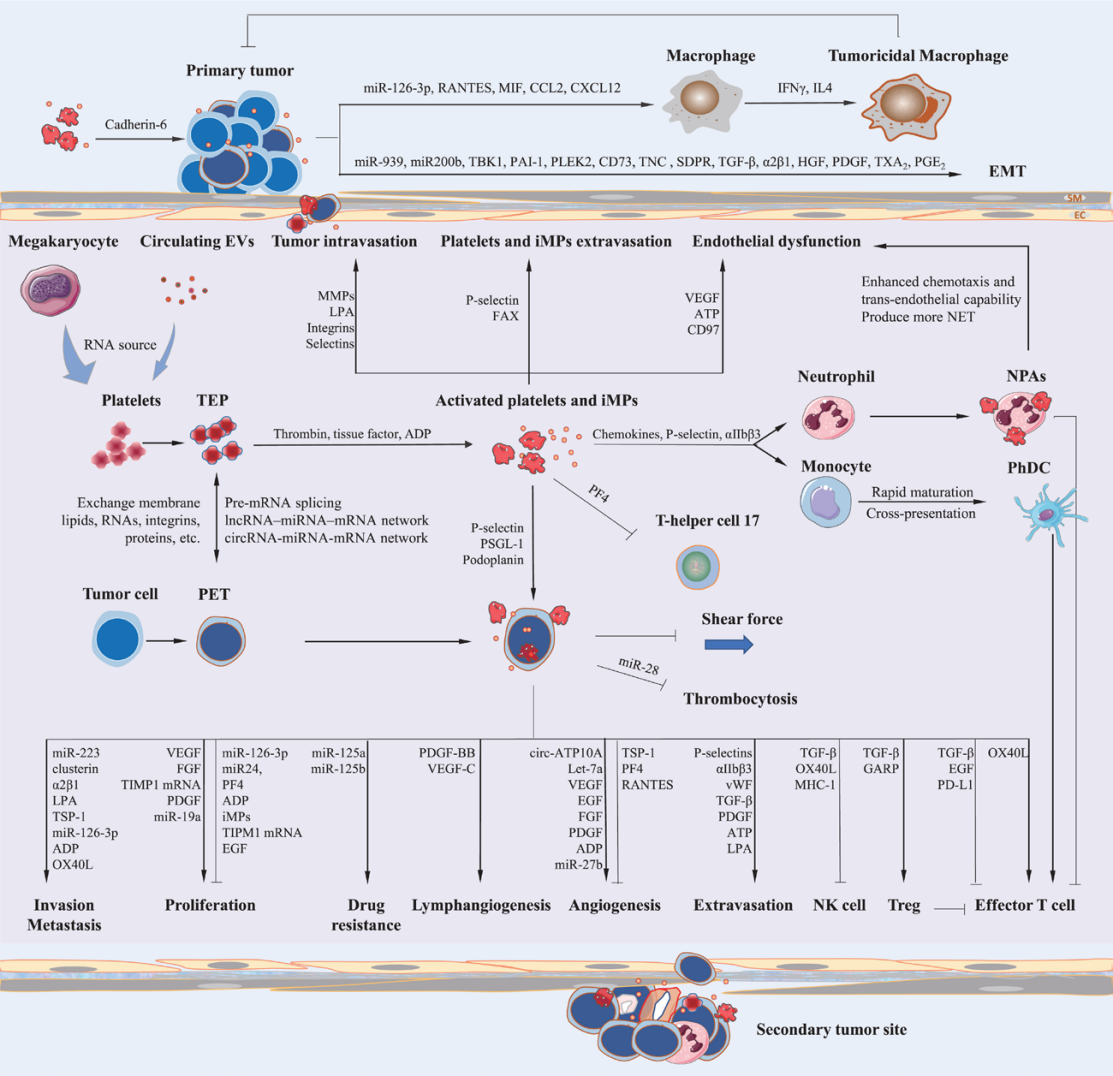
Platelets in the tumor microenvironment. Platelet transcriptome mainly reflects its parental megakaryocyte profile and can also be influenced by extracellular vesicles derived from immune cells and tumor cells in circulation. Mutual “education” between circulating tumor cells and platelets through direct contact and platelet phagocytosis, leads to the exchange of genetic material, proteins, and other bioactive molecules, and the generation of platelet- and tumor cell-interacting microparticles (iMPs) with platelet components, tumor components, or both. Studies have confirmed the existence of de novo protein synthesis in platelets, and changes in splicing factors, long non-coding RNAs, circular RNAs and microRNAs during mutual education with tumor cells alters platelet proteomics. Tumor-derived microvesicles carry soluble procoagulant factors that lead to platelet activation, thereby releasing a range of substances such as growth factors, chemokines, and RNAs that in turn promote or inhibit tumor proliferation, invasion, and metastasis in circulation. Activated platelets and iMPs can also carry bioactive molecules and multiple types of RNA through blood vessels into tumor tissues, interact with tumor cells, promote the recruitment and activation of tumoricidal macrophages, epithelial-mesenchymal transformation of cancer cells, and angiogenesis and lymphangiogenesis in tumor tissues. Moreover, platelet interactions with circulating immune cells and vascular endothelial cells further affect tumor immunity and extravasation. ADP = adenosine diphosphate, ATP = adenosine triphosphate, circRNA = circular RNA, EGF = epidermal growth factor, EMT = epithelial–mesenchymal transition, EV = extracellular vesicles, FAK = focal adhesion kinase protein, FGF = fibroblast growth factor, iMPs = platelet- and tumor cell-interacting microparticles, lncRNA = long non-coding RNA, LPA = lysophosphatidic acid, MHC-1 = major histocompatibility complex-1, miRNA = microRNA, MMP = matrix metalloproteinases, RANTES = Regulated upon Activation Normal T-cell Expressed and Secreted, NK = natural killer, NPAs = neutrophil-platelet aggregates, PDGF = platelet-derived growth factor, PD-L1 = programmed death ligand 1, PET = platelet-educated tumor cells, PF4 = platelet factor 4, PGE_2_ = prostaglandin E_2_, phDC = physiological dendritic cells, PSGL-1 = P-selectin glycoprotein ligand-1, RNA = ribonucleic acid, TBK1 = TANK-binding kinase 1, TEP = tumor-educated platelets, TGF-β = transforming growth factor-β, Treg = regulatory T cell, TSP-1 = thrombospondin 1, TXA_2_ = thromboxane A_2_, VEGF = vascular endothelial growth factor, vWF = von Willebrand factor.

Owing to the smaller size and lower likelihood of being trapped in clots or aggregates, iMPs may more readily exercise the downstream functions of their parental cells. Researchers have found that PMPs can serve as mediators in communication with recipient cells, exerting potent biological effects in the circulatory system, and even infiltrating privileged organs such as the synovium, lymph, and bone marrow.^[22–24]^ Tumor vessels are characterized by high permeability and inadequate perfusion owing to poor pericyte coverage and endothelial dysfunction, which allow the transfer of PMPs and their cargoes to exert pro-tumor or anti-tumor effects.^[12,25]^ Interestingly, this feature of tumor vessels may be driven by vascular endothelial growth factor (VEGF), which was found to be elevated in lung carcinoma cells/platelet co-culture supernatants and may play a key role in lung carcinoma angiogenesis.^[26,27]^ Also, platelets can penetrate tumor vessels and extravasate into the tumor microenvironment, possibly by relying on P-selectin and platelet focal adhesion kinase protein.^[18,21]^ Figure 1 illustrates the key aspects of platelets in cancer progression.

### 2.2. Platelets influence cancer cell behavior via a variety of bioactive components

Platelets are rich in cytokines, chemokines, growth factors, adenosine triphosphate (ATP), enzymes, and attachment factors. Upon activation, platelet α-granules release a wide range of growth factors that have been shown to support the growth and expansion of a variety of cancer cells in vitro, such as platelet-derived growth factor (PDGF), epidermal growth factor (EGF), fibroblast growth factor-β, transforming growth factor-β (TGF-β), hepatocyte growth factor, and VEGF, have been shown to support the growth and expansion of a variety of cancer cells in vitro.^[28]^ It is well known that TGF-β, hepatocyte growth factor, PDGF, thromboxane A_2_, and prostaglandin E_2_, could directly promote or help to trigger EMT and migration in cancer cells.^[29,30]^ TGF-β and PDGF also help to open the capillary endothelium to facilitate cancer cell extravasation from the primary site.^[31,32]^ EGF, PDGF, fibroblast growth factor, ILs, and matrix metalloproteinases (MMPs) are stimulants of tumor angiogenesis, whereas platelet factor 4 (PF4) and regulated upon activation normal t-cell expressed and secreted act as inhibitors.^[33]^ Additionally, PF4 inhibits the IL-17/Stat3 pathway by upregulating the suppressor of cytokine signaling 3, impeding tumor growth in murine melanomamodels.^[34]^ Moreover, PDGF-BB and VEGF-C have been shown to potentiate the proliferation of human lymphoendothelial cells in a dose- and time-dependent manner.^[35]^ Lysophosphatidic acid (LPA) is a bioactive lipid mediator released by platelets that enhances the activity of MMPs. LPA mediates the activation of LPA receptor 1 and serves as a tumor cell mitogen and an accelerator of osteolysis, promoting invasion and metastasis in breast cancer, ovarian cancer, and osteosarcoma.^[36–38]^ More interestingly, researchers have found that platelet-rich EGF induces the upregulation of programmed death ligand 1 in cancer cells in an EGF receptor-dependent manner, revealing a new mechanism of platelets in tumor immune escape.^[39]^ Also, platelet-secreted thrombospondin 1 (TSP-1) and clusterin can stimulate colonic cancer invasion through the signal regulation of MMP-9 via a P38MAPK-regulated pathway.^[40]^ Targeting the CD47/TSP-1 signaling axis in melanoma patients may preserve T cell immunity to lessen tumor burden as monotherapy or in combination with anti-programmed cell death protein 1.^[41]^ However, it is important to note that platelets respond selectively to different stimuli, contributing to the distinct release of pro- or anti-angiogenic components from their α granules.^[42]^ Furthermore, both activated platelets and tumor cells increase the production of adenosine diphosphate, which has extensive physiological effects and plays a contradictory biological role in tumor development, supporting angiogenesis and metastasis, but suppressing cell proliferation.^[43]^

Platelet integrins, glycoproteins, and tumor cell receptors play integral roles in tumor progression and involve a variety of signaling pathways. A human tumor microenvironment chip revealed that the binding of glycoprotein VI to galectin-3 under shear bridges the interaction between platelets and ovarian cancer cells, promoting invasion and proliferation, and RNA sequencing (RNA-seq) confirmed the upregulation of NF-κB, TGFβ/SMAD, EMT pathways, and transfer-regulated signaling pathways, such as the Hippo, MAPK, mTOR, Notch, PI3-Akt, and Wnt pathways in cancer cells induced by platelets.^[44]^ Jia et al demonstrated that platelet activation by a platelet-proteinase-activated receptor-1 agonist triggered TGF-β secretion, which contributed to EMT by downregulating miR-200b.^[45]^ Also, platelet integrin α2β1 contacting promotes the expression of EMT proteins and the autocrine of TGF-β1 in breast cancer cells by activating the Wnt-β-catenin pathway.^[46]^ In addition, αIIbβ3, P-selectins, and ATP released by activated platelets can bind to their corresponding ligands or receptors to facilitate the adhesion of tumor cells to endothelial cells and promote extravasation.^[47,48]^

The binding of platelet P-selectin to P-selectin glycoprotein ligand-1 in lung cancer cells mediates the interaction of activated platelets with cancer cells.^[49]^ Podoplanin is a cell-surface mucin-like glycoprotein that is overexpressed in several tumor cells, as well as in the tumor stroma, inducing platelet aggregation by binding to platelet receptor C-type lectin-like receptor 2 and modulating signal transductions that enhance malignant progression.^[50,51]^ Furthermore, aberrant expression of the podoplanin gene in promyelocytes is the most distinctive transcript of promyelocytic leukemia, and is associated with thrombocytopenia and prolonged bleeding time in a xenograft model.^[52]^ In preclinical models, platelets binding to CD97 overexpressed on tumor cells can autonomously initiate αIIbβ3-dependent platelet activation to release ATP and LPA, and stimulate tumor cell CD97/LPA receptor-dependent Rho activation, resulting in endothelial junction destruction, vascular extravasation and metastasis.^[53]^ Furthermore, Zhang et al found that the atypical IkB serine/threonine kinase TANK-binding kinase 1 expressed in cancer cells mediates the platelet-induced NF-κB signaling pathway and EMT, contributing to cancer invasiveness and may be a driver of breast cancer metastasis.^[54]^

### 2.3. Platelets hop around the tumor microenvironment

A mass of chemokines released by platelets support leukocyte recruitment, and P-selectin has an affinity for both neutrophils and monocytes.^[55]^ The physical interaction of platelets with neutrophils forms neutrophil-platelet aggregates, which produce more neutrophil extracellular traps and exhibit enhanced degranulation, chemotaxis, and trans-endothelial migration features that can be suppressed by αIIbβ3 inhibitors and aspirin.^[56]^ Lecot et al reported that platelets preferentially bind to low-density neutrophils, systemically priming and empowering them to promote cancer progression once they reach the tumor site and endow them with transcriptomic signatures associated with poorer prognosis in patients with pancreatic adenocarcinoma and liver hepatocellular carcinoma.^[57]^ Similarly, platelets interact with monocytes to initiate antigen cross-presentation with cytokine-independent rapid maturation into physiological dendritic cells, which enables more effective tumor-specific T-cell immunity than cytokine-derived dendritic cells.^[58]^

Platelets can transfer high levels of platelet-derived normal major histocompatibility complex-1 to tumor cells with low or absent expression of MCH-1, and induce the release of soluble NKG2D ligand from the cancer cells, interfering with the recognition of cancer cells by natural killer (NK) cells, thus impairing cytotoxicity and IFN-γ production. Mediated by TGFβ, platelets inhibit the expression of CD226 and CD96 on the NK cell surface and their associated ligands on tumor cells to further enhance NK cell suppression.^[59,60]^ TGF-β also regulates the development, homeostasis, tolerance, and immunity of other T cells including CD4^+^ T cells, CD8^+^ T cells, helper T cells 1, helper T cells 2, and cytotoxic T lymphocytes.^[61]^ Additionally, Rittig et al found that in breast cancer patients, the high expression of the platelet immune checkpoint OX40L was not only related to the enhanced activation of T and NK cells but also directly related to the proliferation index of cancer and the formation of metastasis, playing a controversial role in tumor progression.^[62]^

A multiplatform omics analysis revealed that both inflammatory and coagulation pathways as well as resting macrophages and regulatory T cells, were consistently elevated in the venous thromboembolism group of ovarian cancer patients compared to the control group.^[63]^ Furthermore, PMP uptake by primary human macrophages results in the upregulation of several miRNAs, including miR-126-3p, modifying their downstream transcriptome, including mRNAs encoding cytokines/chemokines CCL4, CSF1, and tumor necrosis factor, and reprogramming macrophages into phagocytotic phenotypes.^[64]^ GARP, encoded by the Lrrc32 gene, is a cell surface docking receptor of latent TGFβ, which is abnormally expressed in breast, lung, and colon cancers. GARP can enrich and activate latent TGF-β in the tumor microenvironment to promote FOXP3^+^ regulatory T cell activity, leading to immune tolerance and enhanced cancer progression.^[65]^ However, platelets and PMPs have also been found to suppress the activation of T-helper cell 17 via PF4, promoting cancer proliferation but also boosting IFN-γ production and killing effect on cancer cells.^[66]^

Moreover, RNA, protein, and image analysis of a 3D tetra-culture tissue model indicated that platelet stimulation produces a diseased extracellular matrix in high-grade serous ovarian cancer with the upregulation of EMT and extracellular matrix genes, and platelet activation of mesothelial cells is vital for stimulating cancer cell invasion.^[67]^ The important role of TGF-β and LPA in extracellular matrix remodeling has been demonstrated by researchers. Additionally, platelet integrins and selectins have been shown to support the intravasation of tumors.^[68]^

## 3. Platelet RNAs in tumor progression

Only 70 to 90% of the platelet transcriptome reflects its parental megakaryocyte profile, as platelets can randomly sequester RNA or alter their nucleic acid content in response to external stimuli such as neutrophils, monocytes, tumor cells, or circulating extracellular vesicles from other sources.^[69–72]^ In addition to mRNA, human platelets are rich in diverse miRNAs.^[73]^ Functional Ago2-miRNA complexes can hitch rides on PMPs and heterotypically regulate gene expression in recipient cells.^[74]^ Besides, circRNAs are enriched in platelets by 17- to 188-fold relative to nucleated tissues, whereas exons within circRNAs are enriched by an average of 12.7 times.^[75]^ Interestingly, a selective release of circRNAs into vesicles was noticed by researchers, implicating a specific sorting mechanism that was presumably caused by a response to signals from the environment, just as tumor cells alter the platelet mRNA signature.^[76]^ Researchers have also found differential expression of platelet-associated lncRNAs in cancer, as well as molecular evidence of crosstalk between platelets and serum.^[77–79]^ The interaction between platelets and tumor cells can affect the expression profile of platelets through different mechanisms. Mantini et al investigated the inherent regulation and the potential “education” of platelet RNAs using omics analysis in pancreatic cancer, showing that platelets altered their RNA repertoire via dysregulation of miRNAs and splicing factors, lending color to the existence of de novo protein machinery that causes platelet education.^[80]^ In a mouse model of cancer cell lines of epithelial origin co-cultured with platelets, genes for cell motility, migration, invasion, adhesion, development, differentiation, and inflammation were found to increase after platelet interaction, with 5 commonly altered genes – PAI-1, PLEK2, CD73, TNC, and SDPR – associated with mesenchymal phenotypes.^[81]^ Yu et al provided a novel circRNA-miRNA-mRNA network, suggesting that exosomal circ-ATP10A may promote angiogenesis in multiple myeloma by targeting a set of miRNAs and modulating their downstream mRNAs and could serve as a useful prognostic biomarker.^[82]^ A study of platelet RNA-seq in early nasopharyngeal carcinoma patients demonstrated that SELP was regulated by 5 lncRNAs through 4 corresponding miRNAs, providing new insights into changes in platelet RNA profiles in cancer patients.^[83]^

Conversely, platelets also possess the capacity to transfer functional cytosolic RNAs that can be internalized by recipient cells to exert biological effects.^[84,85]^ Platelets brought TIMP1 mRNA into colorectal cancer cells, where it was translated to proteins and thus potentiate cancer development in vivo and in vitro.^[86]^ Cariello et al demonstrated that platelets from patients with visceral obesity were enriched with miR-19a, which can be induced by colon cancer and promote the growth of colon cancer in a xenograft colon cancer model.^[87]^ Nonetheless, an ectopic pancreatic cancer model provided evidence that platelet miRNAs suppress primary tumor growth by broadly modulating mRNA expression, especially genes involved in EMT.^[88]^ Besides, platelet-derived miR-24 directly targets mitochondrial mt-Nd2, and Snora75 resulting in mitochondrial dysfunction and tumor growth inhibition.^[25]^ The decrease in miR-27b level during platelet activation negatively regulates platelet angiogenic activity by enhancing the de novo synthesis of TSP-1.^[89]^ Furthermore, a negative correlation was found between miR-28 expression and platelet count in myeloproliferative neoplasms, which may be due to the dysregulated interaction between miR-28 and the mRNA of the myeloproliferative leukemia virus oncogene during the proliferative stage of megakaryocytopoiesis.^[90]^

In particular, the role of PMP-delivered miRNAs in tumor progression has attracted considerable attention. PMP can initiate robust capillary-like structure formation in human umbilical vein endothelial cells by directly targeting anti-angiogenic TSP-1 mRNA with Let-7a. This effect of PMP could be eliminated by ribonuclease treatment, suggesting that RNA transfer is a key event.^[91]^ In a study of acute myelogenous leukemia, PMP was internalized by THP-1 cells, leading to the elevation of miR-125a and miR-125b, which may lead to resistance to daunorubicin-induced apoptosis.^[92]^ Additionally, PMP-delivered miR-126-3p lowered AKT2 expression, thus inhibiting proliferation and invasion in either triple-negative or less aggressive luminal A breast cancer subtypes.^[93]^ Tang et al revealed that platelet crosstalk with epithelial ovarian cancer cells via miR-939 carried by PMPs enabled EMT and enhanced cancer progression.^[94]^ Moreover, miR-223 delivered by PMP facilitates lung cancer cell invasion by downregulating the tumor suppressor EPB41L3.^[95]^

Targeting tumor-educated platelets (TEP) molecules and PMPs and enlisting platelets as tools may open new avenues for anti-tumor therapeutics.^[37,44,96]^ Gasperi et al demonstrated that polyunsaturated fatty acids, such as arachidonic acid and docosahexaenoic acid could modulate the delivery of PMP-derived miR-223 and miR-126 into breast cancer cells and, in turn, enhance platelet antitumor activities, including cell cycle arrest, migration inhibition, and sensitivity (Se) to cisplatin.^[97]^ More encouragingly, nanomedicines targeting TEPs and platelet membrane-coated nanomedicines for cancer therapy have been developed with varying degrees of success. With the advantage of homology with platelets and natural affinity for tumor cells, they have great potential to revolutionize the drug delivery strategies currently used.^[32]^ However, considering the diversity of TEP-related molecules and the complexity of their roles in tumor progression, more extensive studies are needed to fully understand their balance in various physiological and pathological processes as fundamental for the development of effective and safe remedies.

## 4. Platelet RNAs are informative on the cancer diagnostics journey

### 4.1. Diagnostic potential of platelet RNAs in neoplasms

Platelet RNA-related diagnostic studies have evolved with a variety of design ideas, optimized methodologies, and expanded applicable areas over the past few years. The classes of platelet RNA used in cancer diagnostic studies have expanded from mRNA to lncRNAs, small nuclear RNAs, snoRNAs, circRNAs, and miRNAs.^[98–100]^ Moreover, the detection methods were extended from quantitative real-time polymerase chain reactions (PCRs) or microarrays to RNA-seq. To learn more about platelet RNAs in cancer screening and diagnosis, related studies published in English up to December 5, 2022, were systematically searched from multiple databases (Cochrane Library, PubMed, Web of Science, Embase, OVID, ScienceDirect, ResearchGate, and Clinical Trials.gov). The inclusion criteria were as follows: all patients were diagnosed based on diagnostic criteria and the sample size was given; control groups were analyzed synchronously; platelet RNA was measured and the method was clearly described; the same outcome: Se, specificity (Sp), area under the curve (AUC), and summary receiver operating characteristic curve; and specimens were limited to platelets isolated from blood. The exclusion criteria were as follows: non-platelet specimens; reviews; abstracts, conference papers, and letters without extractable data; repeated articles; non-human subjects; studies of reanalysis of existing data sets; reports not for validation sets; and articles not published in English. To avoid selection bias, 2 independent reviewers decided whether to include or exclude a study or report only after reaching a consensus. Otherwise, a third reviewer rechecked the process to bridge the differences.

Table 1 details the diagnostic potential of platelet RNAs in neoplasms, while Table 2 displays their diagnostic performance in early-stage neoplasms. Overall, the results of most studies supported the potential of platelet RNAs as markers for cancer diagnosis, but their clinical utility is still controversial. Data from the study by Hänze et al did not support platelet RNAs as a profitable source for early diagnosis of prostate cancer.^[13]^ Moreover, Liefaard et al found that no matter what algorithm was adopted, platelet RNAs cannot be successfully validated in a single-center, independent and blind set of breast cancer, due to the severe variance caused by the hospital of origin, case-control status, and other unknown factors.^[15]^

**Table 1:** Diagnostic potential of platelet RNAs in neoplasms.

Neoplasms	Author	RNA profile	Method	Sample size		Main results
				Patients	Controls	
Non-small cell lung cancer	Best^[7]^	575 genes	RNA-seq	24	16	Se = 95.8%, Sp = 100%, AUC = 0.977
	Best^[101]^	800 genes	RNA-seq	90	40	AUC = 0.95
		1000 genes	RNA-seq	44	44	AUC = 0.93
	Goswami^[102]^	11 genes	qRT-PCR	10	7	AUC = 0.972
	Luo^[77]^	MAGI2-AS3, ZF AS1	qRT-PCR	101	60	For MAGI2-AS3: Se = 78.2%, Sp = 81.7%; for ZF AS1: Se = 81.2%, Sp = 43.3%; for their combination: Se = 87.1%, Sp = 86.7%
	Xing^[103]^	ITGA2B, SELP	qRT-PCR	91	85	For ITGA2B: Se = 91.2%, Sp = 56.5%, AUC = 0.888
				61	51	For SELP: Se = 96.7%; Sp = 43.1%, AUC = 0.716; for their combination, Se = 72.1%; Sp = 90.2%, AUC = 0.846
			ddPCR	60	48	For ITGA2B, Se = 93.2%, Sp = 95.6%, AUC = 0.967; For SELP, Se = 90.0%, Sp = 100%, AUC = 0.956
	Dong^[104]^	SNORD55	qRT-PCR	290	189	Se = 79.3%, Sp = 68.3%, AUC = 0.803
	D’Ambrosi^[99]^	circNRIP1	qRT-PCR	23	24	circNRIP1 in NSCLC downregulated (*P* = .0302)
	In ’t Veld^[105]^	493 genes	RNA-seq	522	723	AUC = 0.94
Lung cancer	Dong^[98]^	snRNA U1, U2, U5	qRT-PCR	382	361	AUC for U1, U2, U5 and their combination: 0.769, 0840, 0.809, and 0.840
	Li^[78]^	linc-GTF2H2-1, RP3-466P17.2, lnc-ST8SIA4-12	qRT-PCR	167	202	AUC for linc-GTF2H2-1, RP3-466P17.2, lnc-ST8SIA4-12 and their combination: 0.781, 0.788, 0.725, and 0.921
	Liu^[106]^	MAX, MTURN, HLA-B	qPCR	127	82	Se = 60.6%, Sp = 81.7%, AUC = 0.734
Breast cancer	Best^[7]^	192 genes	RNA-seq	16	10	Se = 100%, Sp = 100%, Ac = 100%
	Liefaard^[15]^	NA	RNA-seq	77	62	Internal validation set: AUC of 0.86 for PSO-SVM; AUC of 0.87 for elastic net algorithm
				37	36	Single-center, independent, blinded set: AUC of 0.57 for the PSO-SVM, AUC of 0.60 for elastic net algorithm
	Yao^[107]^	TPM3	qRT-PCR	504	109	AUC of 0.9705 for diagnosis, AUC of 0.8404 for metastatic cancer
	In ’t Veld^[105]^	493 genes	RNA-seq	93	723	AUC = 0.81
	Lips^[108]^	NA	RNA-seq	NA	NA	Validation set: Ac = 86%, AUC = 0.93. For stage I/II patients: Ac = 91%. For rule-out application: Se = 94%, Sp = 67%. For screening setting: Se = 80%, Sp = 93%
				NA	NA	Distinguish breast cancer from other tumors (n = 192, Ac = 87%, AUC = 0.91)
Hepatobiliary cancer	Best^[7]^	59 genes	RNA-seq	6	16	Se = 67%, Sp = 100%, Ac = 91%
Hepatocellular carcinoma	Asghar^[109]^	TGF-β, NFκβ, AKT, PI3K, VEGF	qRT-PCR	20	10	Se = 100%, AUC = 1.0
	In ’t Veld^[105]^	493 genes	RNA-seq	23	723	AUC = 0.96
	Zhu^[110]^	miR-495-3p	qRT-PCR	25	25	AUC = 0.76
		miR-1293	qRT-PCR	25	25	AUC = 0.78
Colorectal cancer	Best^[7]^	1048 genes	RNA-seq	16	16	Se = 100%; Sp = 100%; Ac = 100%
	Xu^[111]^	921 genes	RNA-seq	68	52	Se = 88.5%; Sp = 86.8%; Ac = 87.5%; AUC = 0.920
	Ye^[79]^	LNCAROD, SNHG20, LINC00534, and TSPOAP-AS1	qRT-PCR	105	105	The AUC of LNCAROD, SNHG20, LINC00534, TspoAP-AS1 and their combination were 0.74, 0.73, 0.73, 0.63, and 0.78
	Yang^[86]^	TIMP1	qRT-PCR	286	41	AUC = 0.9583
	In ’t Veld^[105]^	493 genes	RNA-seq	85	723	AUC = 0.88
Glioblastoma	Best^[7]^	60 genes	RNA-seq	16	16	Se = 69%, Sp = 100%, Ac = 84%
	Sol^[112]^	457 genes	RNA-seq	39	52	Ac = 94%, AUC = 0.982
	Sol^[113]^	200 genes	RNA-seq	34	306	Ac = 95%, AUC = 0.97
		212 genes	RNA-seq	23	46	Differentiate from multiple sclerosis and brain metastasis: Ac = 80%, AUC = 0.81
	In ’t Veld^[105]^	493 genes	RNA-seq	132	723	AUC = 0.87
Pancreatic cancer	Best^[7]^	665 genes	RNA-seq	14	16	Validation set: Se = 100%, Sp = 100%, Ac = 100%
	Boyd^[114]^	NA	RNA-seq	112	60	Se = 71%, Sp = 98%, AUC = 0.93.
	Large^[115]^	NA	RNA-seq	135	322	Controls: 250 healthy controls and 72 benign pancreatobiliary disease patients: Ac >90%
	In ’t Veld^[105]^	493 genes	RNA-seq	126	723	AUC = 0.81
Nasopharyngeal carcinoma	Sun^[116]^	miR-18a-3p	qRT-PCR	54	36	Se = 87%, Sp = 72.7%, AUC = 0.841
	Wang^[100]^	miR-34c-3p	qRT-PCR	54	36	Se = 94.44%, Sp = 86.11%, Ac = 91.11%, AUC = 0.952
		miR-18a-5p		54	36	Se = 85.19%, Sp = 86.11%, Ac = 85.55%, AUC = 0.884
		miR-34c-3p + miR-18a-5p		54	36	Se = 92.59%, Sp = 86.11%, Ac = 90.00%, AUC = 0.954
Myeloproliferative neoplasms	Gorbenko^[117]^	JAK2 V617F allele burden	qRT-PCR	24	–	Correlation between leukocyte DNA and platelet mRNA: *R* = 0.79, *R*^2^ = 0.62; changes in platelet RNA preceded changes in leukocyte DNA
	Shen^[118]^	3326 genes	RNA-seq	95	21	The progressive transcriptome model in the classification of 24 essential thrombocythemias, 33 polycythemia vera, and 40 myelofibrosis: AUC = 0.97
Thyroid neoplasm	Shen^[119]^	765 genes	RNA-seq	42	27	Ac = 97%; AUC = 0.998. The average Ac for the diagnosis of thyroid adenomas, papillary thyroid cancer and metastasized papillary thyroid cancer was 80.5%
Prostate cancer	Hänze^[13]^	PCA3	qRT-PCR	31	29	Without significant differences
		MALAT1, EZH2, AMACR, PSGR, PSA, PSMA, TRPM8	qRT-PCR	23	21	Without significant differences
	In ’t Veld^[105]^	493 genes	RNA-seq	35	723	AUC = 0.98
Sarcoma	Heinhuis^[120]^	2647 genes	RNA-seq	17	36	Se = 88%, Sp = 86%, AUC = 0.93
	In ’t Veld^[105]^	493 genes	RNA-seq	53	723	AUC = 0.96
Renal cell carcinoma	Xiao^[121]^	68 genes	RNA-seq	24	25	Se = 91.7%, Sp = 100%, Ac = 95.9%, AUC = 0.988
	In ’t Veld^[105]^	493 genes	RNA-seq	28	723	AUC = 0.87
Esophageal carcinoma	Liu^[122]^	ARID1A, GTF2H2, PRKRIR	RNA-seq	48	53	Se = 87.5%, Sp = 81.1%, AUC = 0.893
	In ’t Veld^[105]^	493 genes	RNA-seq	15	723	AUC = 0.8
Melanoma	In ’t Veld^[105]^	493 genes	RNA-seq	68	723	AUC = 0.9
Multiple myeloma	In ’t Veld^[105]^	493 genes	RNA-seq	31	723	AUC = 0.99
Lymphoma	In ’t Veld^[105]^	493 genes	RNA-seq	20	723	AUC = 0.92
Ovarian cancer	In ’t Veld^[105]^	493 genes	RNA-seq	144	723	AUC = 0.89
Cholangiocarcinoma	In ’t Veld^[105]^	493 genes	RNA-seq	85	723	AUC = 0.91
Endometrial cancer	In ’t Veld^[105]^	493 genes	RNA-seq	39	723	AUC = 0.78
HNSCC	In ’t Veld^[105]^	493 genes	RNA-seq	101	723	AUC = 0.92
Urothelial carcinoma	In ’t Veld^[105]^	493 genes	RNA-seq	28	723	AUC = 0.91

– = not applicable, Ac = accuracy, AUC = area under the curve, DNA = deoxyribonucleic acid, HNSCC = head and neck squamous cell carcinoma, mRNA = messenger ribonucleic acid, NA = not available, NSCLC = non-small cell lung cancer, PSA = prostate-specific antigen, qPCR = quantitative polymerase chain reaction, qRT-PCR = quantitative real-time PCR, RNA = ribonucleic acid, RNA-seq = RNA sequencing, Se = sensitivity, snRNA = small nuclear RNA, Sp = specificity, TGF-β = transforming growth factor-β, VEGF = vascular endothelial growth factor.

**Table 2:** Diagnostic potential of platelet RNAs in early-stage neoplasms.

Neoplasms	Author	RNA profile	Method	Sample size		Main results
				Patients	Controls	
Non-small cell lung cancer	Xing^[103]^	ITGA2B	qRT-PCR	56	206	Se = 87.8%, Sp = 56.5%, AUC = 0.842
		SELP	qRT-PCR	56	206	Se = 93.1%, Sp = 43.1%, AUC = 0.642
	Dong^[104]^	SNORD55	qRT-PCR	91	189	Se = 91.2%, Sp = 49.7%, AUC = 0.784
	Best ^[101]^	1000 genes	RNA-seq	53	53	Se = 81.3%, Sp = 75.5%, AUC = 0.890
Lung cancer	Liu^[106]^	MAX, MTURN, HLA-B	qPCR	33	82	Se = 72.7%, Sp = 85.4%, AUC = 0.787
	Li^[78]^	linc-GTF2H2-1, RP3-466P17.2, lnc-ST8SIA4-12	qRT-PCR	47	202	AUC for linc-GTF2H2-1, RP3-466P17.2, lnc-ST8SIA4-12 and their combination: 0.704, 0.771, 0.768, and 0.895
	Dong^[98]^	snRNA U1, U2, U5	qRT-PCR	80	361	AUC for U1, U2, U5 and their combination: 0.669, 0.805, 0.752, and 0.826
Breast cancer	Liefaard^[123]^	NA	RNA-seq	NA	144	Discriminate from noncancer: AUC = 0.72
				NA	NA	Discriminate from other tumor: AUC = 0.78
Hepatocellular carcinoma	Asghar^[109]^	PI3K, AKT	qRT-PCR	12	10	AUC were 0.9286 and 0. 8751, respectively
	Waqar^[124]^	CTNNB1, SERPIND1, SPINK1	qRT-PCR	20	20	Differentiating from late-stage cirrhotic nodules: the AUC of CTNNB1, SERPIND1, SPINK1 and their combination for 0.94, 0.88, 0.89, and 1.0 respectively
Pancreatic cancer	Boyd^[114]^	NA	RNA-seq	NA	NA	Differentiate from late stages (AUC = 0.91)
	Large^[115]^	NA	RNA-seq	NA	NA	Classified early-stage patients correctly and distinguished them from advanced and benign diseases
MGUS	Van Eijs^[125]^	1371 genes	RNA-Seq	24	29	Differentiate from healthy controls (*P* < .0001)
Melanoma	Yin^[126]^	36 gene	RNA-seq	–	–	Discriminate mice with occult tumors from controls: AUC = 0.912. Discriminate from macroscopic tumor: AUC = 0.936

– = not applicable, AUC = area under the curve, MGUS = monoclonal gammopathy of undetermined significance, NA = not available, qPCR = quantitative polymerase chain reaction, qRT-PCR = quantitative real-time PCR, RNA = ribonucleic acid, RNA-seq = RNA sequencing, Se = sensitivity, Sp = specificity.

In some studies, the diagnostic performance of combinated RNAs was superior to that of individual RNA, suggesting that using platelet panels as diagnostic tools may be preferable.^[78,98,103]^ To identify eligible RNA panels, diverse screening algorithms and machine learning methods have been employed, which may influence the evaluation of diagnostic value.^[102,127,128]^ In a study by the Goswami team, the diagnostic performance of 11 gene panels was the best under the application of gradient boosting, followed by Random Forests, and the worst under linear discriminant analysis. However, in a report of 1000 gene panels, the linear discriminant analysis yielded the highest AUC, indicating that the best-matched classification methods for different numbers of genes vary.^[102]^ Also, the Kyoto Encyclopedia of Genes and Genomes pathway applied in the screening strategy may contribute to the acquisition of clinically significant platelet RNAs modulated by tumor cells and improve diagnostic performance.^[128,129]^

Currently, there are few studies on snoRNAs, lncRNAs, snRNAs, and other non-coding RNAs, and their practical value in cancer diagnosis requires further investigation. Some studies have limitations regarding literature quality, such as small sample size, unclear study type, and lack of suspected patients in the control group, which may have undermined the evaluation of platelet RNA. In addition, some studies provided summary receiver operating characteristic curves and AUC values without Se and Sp data, which may not be conducive to further systematic review because the determination of the cutoff value is not universal even for a certain diagnostic test and should be determined according to the region and disease situation.^[130]^

### 4.2. Platelet RNAs for monitoring progression and guiding treatment in neoplasms

The time lag between tumor progression or drug resistance and significant symptoms or substantial changes in imaging may lead to the loss of the best opportunity for patients, which in turn may affect therapeutic efficacy or lead to poor outcomes.^[131]^ Monitoring tumor progression and guiding treatment calls for sensitive and reliable markers, and the role of platelet RNAs in tumor development suggest that they may be useful. Ge et al discovered many differentially expressed mRNAs in platelets between patients with localized and metastatic cancer, and a few genes showed an upward trend from the early to late stage of cancers.^[132]^ Also, Liu et al found that ARID1A tended to have a positive correlation with the size and stage of esophageal squamous cell carcinoma, whereas GTF2H2 and PRKRIR tended to have a negative correlation. Additional information on platelet RNAs for monitoring cancer progression is presented in Table 3.

**Table 3:** Platelet RNA in monitoring cancer progression.

Neoplasms	Author	RNA profile	Method	Sample size		Main results
				Patients	Controls	
Non-small cell lung cancer	Best^[101]^	1000 genes	RNA-seq	245	273	For metastasized late-stage: AUC = 0.940
	Ge^[132]^	FCGR2A, KLHDC8B, IGFBP2, ARL2	RNA-seq	345	234	For metastatic cancer: AUC were 0.705, 0.707, 0.711 and 0.791, respectively
Lung cancer	Li^[78]^	linc-GTF2H2-1	qRT-PCR	235	–	For differentiating 235 advanced-stage from 80 early-stage lung cancer: AUC = 0.645
Hepatocellular carcinoma	Asghar^[109]^	AKT, PI3K	qRT-PCR	NA	NA	Increased with the advancement of cancer stage
Colorectal cancer	Ye^[79]^	LNCAROD	qRT-PCR	NA	NA	Higher expression of LNCAROD associated with advanced cancer (stage III/IV) (*P* < .05)
Breast cancer	Yao^[107]^	TPM3	qRT-PCR	49	42	Upregulated and correlated with metastasis. AUC = 0.8404
Esophageal squamous cell carcinoma	Liu^[122]^	ARID1A, GTF2H2, PRKRIR	RNA-Seq	71	80	ARID1A tended to have a positive correlation with tumor size and stage while GTF2H2 and PRKRIR tended to have a negative correlation
Nasopharyngeal carcinoma	Xu^[83]^	SELP	RNA-Seq	22	11	Gradually increases from the early to the advanced stage

– = not applicable, AUC = area under the curve, NA = not available, qRT-PCR = quantitative real-time polymerase chain reaction, RNA = ribonucleic acid, RNA-seq = RNA sequencing.

Platelet RNAs have also displayed potential in identifying driver genomic alterations, monitoring responses, and predicting outcomes of therapeutic regimens, opening up new opportunities for precision medicine. A typical example is that studies have demonstrated that EML4-Alk rearrangement detection in platelets is significantly more sensitive than that in plasma and formalin-fixed paraffin-embedded tissues, with the capacity to predict outcomes in non-small-cell lung cancer patients treated with ALK inhibitors.^[133,134]^ More encouragingly, serial monitoring of EML4-ALk rearrangements in platelets could reveal crizotinib resistance 2 months before radiographic disease progression, representing the clonal evolution of acquired mutations during treatment that causes chemoresistance and guiding the selection of subsequent therapeutic strategies.^[133]^ More information on platelet RNA in cancer prognosis and therapy is summarized in Table 4.

**Table 4:** Platelet RNAs in cancer prognosis and therapy.

Neoplasms	Author	RNA	Method	Main results
Non-small cell lung cancer	Nilsson^[133]^	EML4-ALK rearrangement	qRT-PCR	In 77 patients and healthy donors: Se = 65%, Sp = 100%. 29 EML4-ALK + patients versus 14 EML4-ALK- patients treated with crizotinib and followed for 13 mo: PFS: HR = 3.5, *P* = .02; OS: HR = 3.0, *P* = .11. Revealed drug resistance 2 mo before radiographic disease progression
	Xing^[103]^	ITGA2B	qRT-PCR	Patients with higher ITGA2B versus patients with lower ITGA2B (OS: HR = 2.3, *P* = .005)
		SELP	qRT-PCR	*P* = .893
	Chang^[135]^	PD-L1	–	19 patients (44.4%) with high PD-L1 responded to immunotherapy compared to 5 patients (13.9%) with low PD-L1(*P* < .01). PFS for 64 patients received only immunotherapy, low PD-L1 group versus high PD-L1 group: 2.76 vs 8.02 mo, *P* = .002
	Park^[134]^	ALK rearrangement	qRT-PCR	In 33 FISH-positive and 28 FISH-negative patients: positivity = 87.0%. In 26 patients treated with crizotinib, the platelet-positive subgroup displayed longer PFS (5.7 vs 1.7 mo), higher OS rate (70.6% vs 11.1%), and disease control rate (88.2% vs 44.4%) than the negative subgroup
Lung cancer	Dong^[98]^	U1, U2, U5	qRT-PCR	U1, U2 and U5 were downregulated after anticancer therapy (*P* were 0.0495, 0.0098 and 0.0276, respectively)
	Liu^[106]^	MAX, MTURN, HLA-B	qRT-PCR	Low mRNA expression of these genes was correlated with a “favorable” first chemotherapy response (*P* were 0.0332, 0.0482 and 0.0266, respectively)
Nasopharyngeal carcinoma	Sun^[116]^	miR-18a-3p	qRT-PCR	miR18a-3p were downregulated after 7 of 9 chemotherapy, in 2 of 3 patients
Glioblastoma	Sol^[113]^	TEP score	RNA-seq	Patients before tumor resection compared with those collected during the follow-up period (0.91 vs 0.44, *P* < 2.2 × 10^−16^). Correlation between TEP score and OS in the first postoperative sample: *R* = 0.33, *P* = .03, n = 30.
Prostate cancer	Hänze^[13]^	PSA	qRT-PCR	PSA-mRNA was detected in 2 cases (10%) but not in controls and could be an indicator of a less favorable prognosis and early relapse
Acute myelocytic leukemia	Bao^[136]^	ATF4	RNA-seq	ATF4 was upregulated in the elderly and has been reported to promote hematopoietic stem cells survival, guiding different strategies for therapy

– = not applicable, HR = hazard ratio, mRNA = messenger ribonucleic acid, OS = overall survival, PD-L1 = programmed death ligand 1, PFS = progression-free survival, PSA = prostate-specific antigen, qRT-PCR = quantitative real-time polymerase chain reaction, RNA = ribonucleic acid, RNA-seq = RNA sequencing, Se = sensitivity, Sp = specificity, TEP = tumor-educated platelets.

### 4.3. Recommendations for platelet RNA-related diagnostic studies

To achieve the generalizability of platelet RNA studies in cancer and other fields, standardization of platelet isolation, sequencing, and machine-learning algorithms, as well as the dissemination of bioinformatic codes, have been suggested.^[137,138]^ Based on the characteristics of existing platelet RNA-related diagnostic studies, we propose the following points from a clinical perspective, hoping to pave the way for future researchers to identify and control potential variables throughout the studies to reach practical conclusions.

Patients and controls: platelet RNA landscape may vary with treatment regimens and duration, tumor stages and histological types, so “newly diagnosed with clear diagnostic criteria or untreated patients” should be set as one of the inclusion criteria, and the stage and typing information of cases should be provided in detail and the corresponding platelet RNA panels should be explored.^[79,119,129]^ To avoid overestimating the diagnostic value, in addition to healthy donors, the control group should include suspected cases, such as the precursor stage of cancer, benign disease, and inflammatory cases, from which malignant disease is more difficult to discriminate.^[10,139]^ Moreover, sex, age, obesity, smoking status, etc might affect the platelet RNA profile and should be properly matched with the experimental groups.^[87,98,140,141]^ Furthermore, researchers found that the performance of platelet RNAs was not desirable in differentiating breast cancer from other cancers, suggesting that the inclusion of patients with other cancers is necessary to improve Sp during the screening phase.^[123]^

Study design: To avoid overrating diagnostic performance, case-control studies are inadvisable, and cohort studies may be more applicable initially, considering the limited source.^[15]^ After the diagnostic power has been preliminarily qualified, a randomized controlled trial should be conducted for further confirmation. In addition, given the different Se and Sp requirements of cancer screening and confirmation tests, screening and confirmation cohorts should be established separately, and more suitable RNA panels should be selected.^[108,123]^

Detection strategy: Platelet sample preparation, RNA detection, and bioinformatics analysis should be standardized, unified, and evolved based on the discovery of influencing factors. In the candidate RNA selection phase, RNA-seq might be more applicable than quantitative PCR because of its ability to map the RNA landscape in patients, such as driver mutations and differential alternative splicing. Additionally, the application of gene databases, such as Gene Ontology or Kyoto Encyclopedia of Genes and Genomes pathway, as well as advanced deep learning methods facilitates the screening of core genes with high Ac, clinical relevance, and cost-effectiveness.^[142]^ However, in the validation phase, the insufficient Se and precision of RNA-seq for specific genes might lead to biased results, whereas quantitative PCR with a rigorous stepwise process or digital PCR could provide precise and quantitative data.^[103,143–146]^

Sample size: To ensure the Ac of the algorithm, a relatively large number of real samples is necessary, which can be achieved through cross-institutional collaborations.^[119]^ In some existing studies, the EigenSample technique was adopted to augment the data, which may have biased the evaluation results to some extent.^[102,147]^

Primary data and diagnostic parameters: Determination of diagnostic parameters requires strict adherence to the principle of blindness, and each parameter should be documented in detail. A shared database of platelet RNAs and detailed demographic and clinical characteristics spanning both healthy individuals and patients with different diseases, including neoplasms, is urgently needed to explore the clinical value of platelet RNA.

## 5. Conclusion

Taken together, exploring the activities of platelets and their RNAs in the tumor microenvironment provides deeper insights into the mechanisms of cancer progression as well as more opportunities for targeted treatment. With the assistance of well-designed screening algorithms, platelet RNAs perform well in the diagnosis and early screening of neoplasms. Moreover, their potential for cancer prognosis and guiding treatment is noticeable. However, obtaining clinically substantial results in platelet RNA-related diagnostic studies depends heavily on the Ac and precision of the detection method as well as the standardization and normalization of variable control in pre-analytical, analytical, and post-analytical processes. Large-scale, multicenter, well-designed studies are warranted to advance platelet RNAs for clinical cancer management and benefit patients.

## Acknowledgments

We sincerely appreciate the efforts of researchers and volunteers to investigate the role of platelets in neoplasms.

## Author contributions

**Conceptualization:** Yunhui Xiang, Yanying Li, Juan Zhang.

**Data curation:** Yunhui Xiang, Pinpin Xiang, Liuyun Zhang, Yanying Li.

**Formal analysis:** Yunhui Xiang, Pinpin Xiang.

**Funding acquisition:** Juan Zhang.

**Validation:** Yunhui Xiang, Pinpin Xiang, Yanying Li, Juan Zhang.

**Visualization:** Yunhui Xiang, Pinpin Xiang.

**Writing – original draft:** Yunhui Xiang, Pinpin Xiang, Liuyun Zhang, Yanying Li.

**Writing – review & editing:** Yunhui Xiang, Pinpin Xiang, Yanying Li, Juan Zhang.
